# Supplementary material for: AS-Quant: Detection and Visualization of Alternative Splicing Events with RNA-seq Data
Source: Int J Mol Sci. 2021 Apr 25;22(9):4468. doi: 10.3390/ijms22094468 (PMC8123109; doi:10.3390/ijms22094468)
Supplement: Supplementary file 1 [file ijms-22-04468-s001.zip › ijms-1169729-SI.pdf]

AS-Quant: Detection and Visualization of Alternative Splicing Events with RNA-seq Data

Naima Ahmed Fahmi, Heba Nassereddeen, Jae-Woong Chang, Meeyeon Park, Hsin-Sung Yeh, Jiao Sun, Deliang Fan, Jeongsik Yong, and Wei Zhang

April 19, 2021

1 Parameters to run flux-simulator (50 million reads)

Parameters	Value	Description
REF_FILE_NAME	mm10_ucsc.gtf	GTF reference annotation
GEN_DIR	Genome_mm10	Genomic sequences directory
NB_MOLECULES	10000000	Number of RNA molecules
TSS_MEAN<br>POLYA_SCALE<br>POLYA_SHAPE	100<br>100<br>2	Transcript modification parameters
FRAG_SUBSTRATE<br>FRAG_METHOD<br>FRAG_NB_LAMBDA<br>FRAG_NB_M	DNA<br>NB<br>575<br>1	Library Preparation parameters
RTRANSCRIPTION	YES	Switch on reverse transcription
PCR_DISTRIBUTION<br>GC_MEAN<br>GC_SD<br>PCR_PROBABILITY	none<br>NaN<br>NaN<br>0.1	Amplification parameters
FILTERING	YES	Switches size selection On
UNIQUE_IDS	TRUE	Create Unique Read Identifiers for paired-end
READ_NUMBER	50000000	Number of reads
READ_LENGTH	76	Length of each read
PAIRED_END	YES	Paired end reads
FASTA	YES	Generate Fasta file
ERR_FILE	76	Error model for length 76

2 Realtime quantitative PCR (RT-qPCR) analysis and primer sequences

The primer sequences used to measure the expression for transcript isoforms for the genes are following:
mPtbp1 forward: 5'-TGCAGTATGCTGACCCTGTG-3'
mPtbp1 reverse: 5'-AGCTGCACACTCTGATGCTT-3'
mGanab forward: 5'-GATCGATGAGCTAGAGCCCC-3'
mGanab reverse: 5'-TCCAAACCTACAGACGTGGG-3'
Tpm3 forward: 5'-CCGATAGGAAGTATGAAGAGGTGGCTCGTAAG-3'
Tpm3 reverse: 5'-GTCCAGCATCCTTTGTGTACAGAGATGCTC-3'
Camk2g forward: 5'-GAGAAAACTGAAGGGTGCCATCCTCACAAC-3'
Camk2g reverse: 5'-GTCACAAATCTTCGTGTAGGCCTCAAAGTCC-3'

3 Commands for running baselines

3.1 SUPPA2

Input:

1. Annotation in GTF format: annotation.GTF
2. Event files in .ioe format, generated from the annotation for all five types of alternative splicing and merged them into *annotation.events.ioe*
3. transcript expression files (.tpm) for all six samples (an example) in both conditions.

Step 1: Generate event files (input preparation)
$ python3 suppa.py generateEvents –pool-genes -i annotation.GTF -o out_file -f ioe -e SE SS MX RI

Step 2: Prepare transcript expression files (input preparation)
Generate .tpm file for each sample in both conditions, and concatenate all case samples into all_case_samples.tpm, and all control samples into all_control_samples.tpm

Step 3: Get PSI files for both case and control
$ python3 suppa.py psiPerEvent -i annotation.events.ioe -e all_case_samples.tpm -o case_PSI
$ python3 suppa.py psiPerEvent -i annotation.events.ioe -e all_control_samples.tpm -o control_PSI

Step 4: Differential splicing analysis
$ python3 suppa.py diffSplice -m empirical -i annotation.events.ioe -p case_PSI.psi control_PSI.psi -e all_case_samples.tpm all_control_samples.tpm –area 500 –lower-bound 0.05 -me -th 1 -o suppa2_out

3.2 rMATS

Input: .bam files with 3 replicates (an example) in each group of sequences

$ python RNASeq-MATS.py -b1 control_seq1.bam,control_seq2.bam,control_seq3.bam
-b2 case_seq1.bam,case_seq2.bam,case_seq3.bam -t paired -len 76 -gtf gtf_name -o rMATS_out_dir

3.3 diffSplice

Input: .sam file with 3 replicates (an example) in each group

Step 1: Update datafile.cfg in diffSplice directory
g1 id1 s1 control_seq1.sam
g1 id1 s2 control_seq2.sam
g1 id1 s3 control_seq3.sam
g2 id1 s1 case_seq1.sam
g2 id1 s2 case_seq2.sam
g2 id1 s3 case_seq3.sam

Step 2: Run diffSplice command
$ diffsplice settings.cfg datafile.cfg diffSplice_dir

4 AUC plots for all methods with different types of alternative splicing events

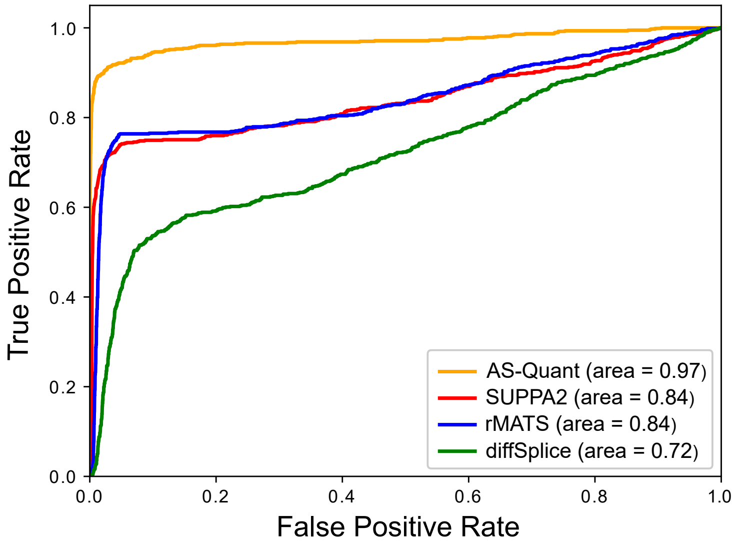

Figure S1: **Simulation experiment (50M reads) to assess the performance of AS-Quant and baseline methods to detect SE events.** The receiver operating characteristic (ROC) curves, i.e., true positive rate against false positive rate, are plotted.

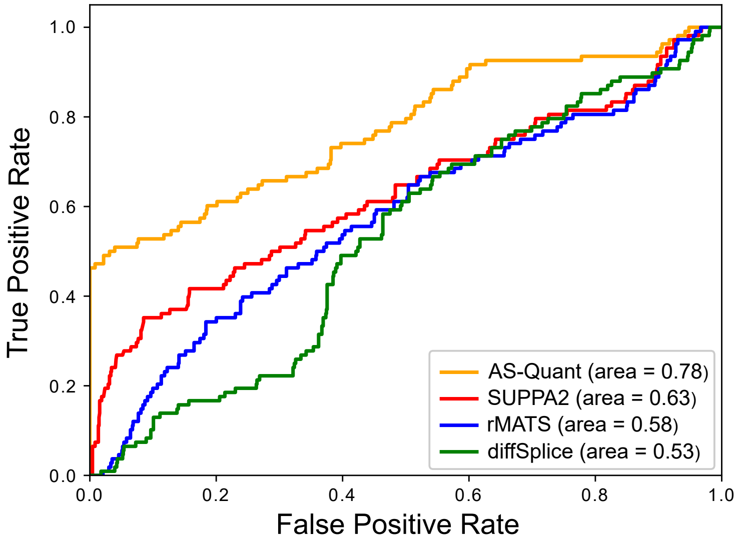

Figure S2: **Simulation experiment (50M reads) to assess the performance of AS-Quant and baseline methods to detect RI events.** The receiver operating characteristic (ROC) curves, i.e., true positive rate against false positive rate, are plotted.

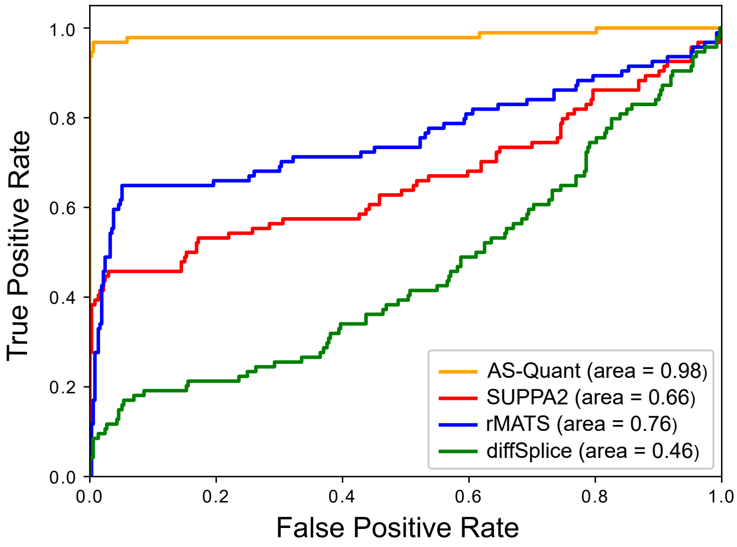

Figure S3: **Simulation experiment (50M reads) to assess the performance of AS-Quant and baseline methods to detect MXE events.** The receiver operating characteristic (ROC) curves, i.e., true positive rate against false positive rate, are plotted.

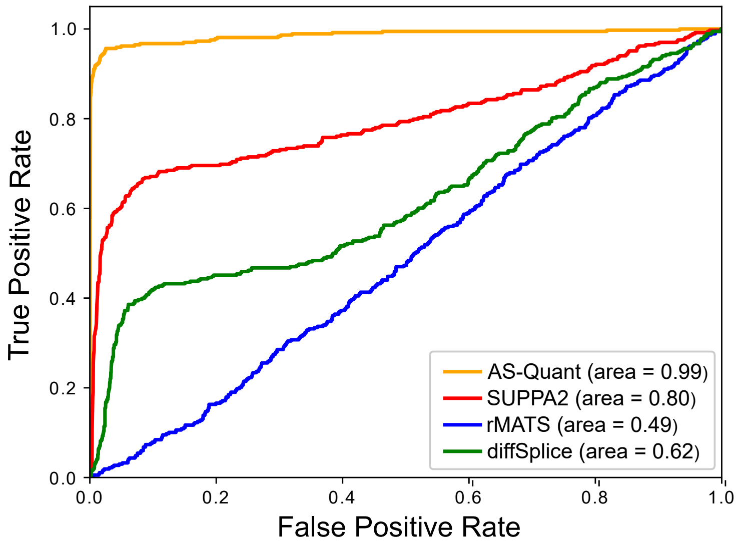

Figure S4: **Simulation experiment (50M reads) to assess the performance of AS-Quant and baseline methods to detect A3SS events.** The receiver operating characteristic (ROC) curves, i.e., true positive rate against false positive rate, are plotted.

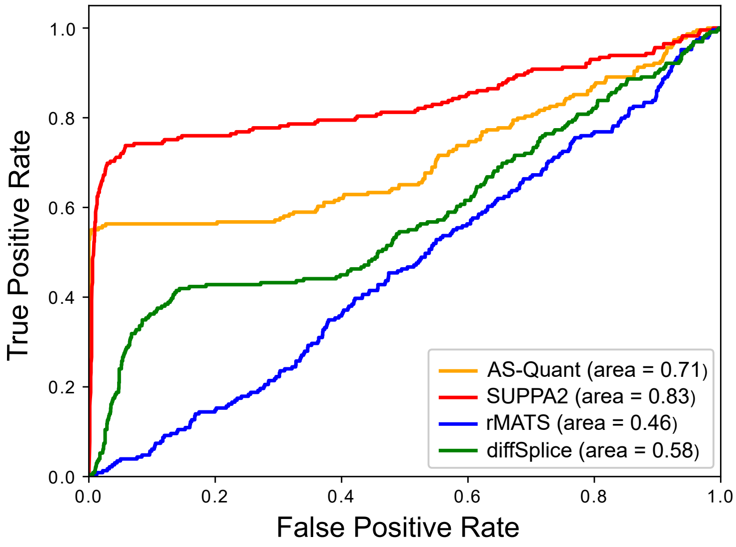

Figure S5: **Simulation experiment (50M reads) to assess the performance of AS-Quant and baseline methods to detect A5SS events.** The receiver operating characteristic (ROC) curves, i.e., true positive rate against false positive rate, are plotted.

5 Unannotated alternative splicing event (SE) detected by AS-Quant

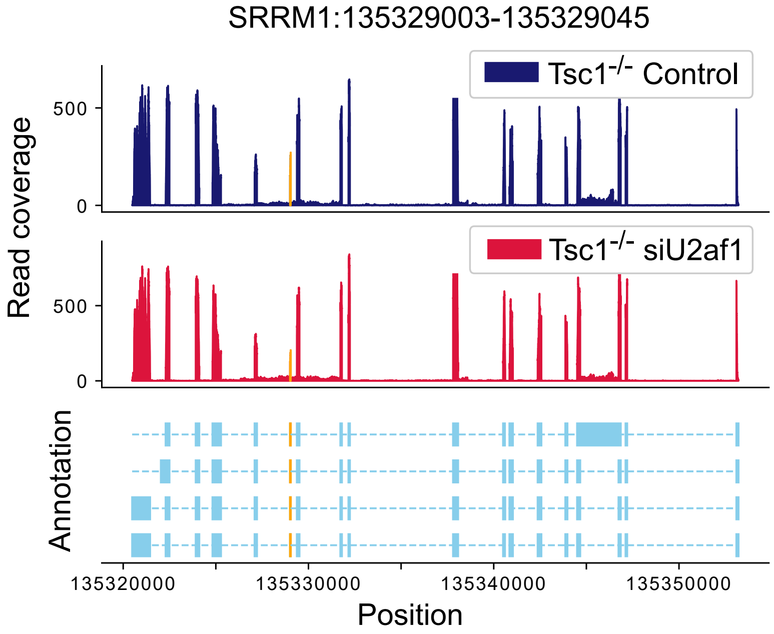

Figure S6: Novel AS event in *SRRM1* identified by AS-Quant. The highlighted exon was not an annotated splicing exon.

6 Intron Retention (RI) event generated bt AS-Quant plot

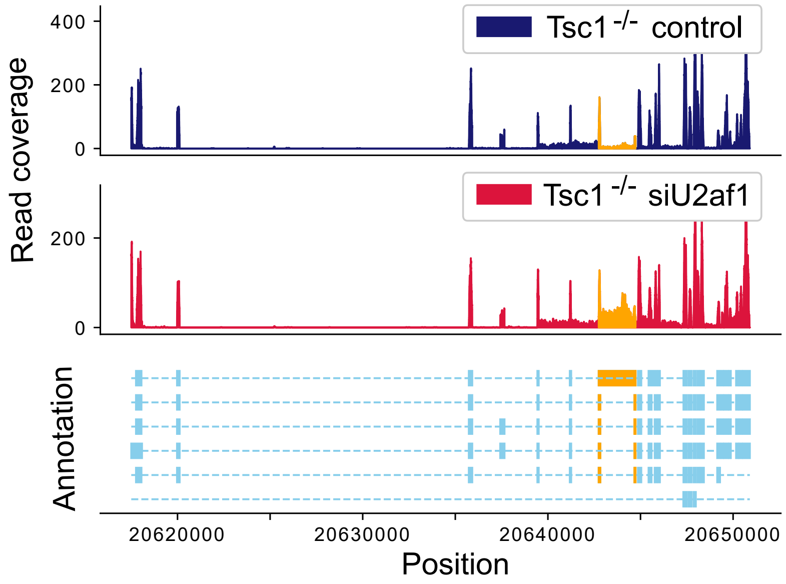

Figure S7: Intron Retention (RI) event in gene *Rbm10* identified and plotted by AS-Quant.

AS-Quant User Manual

1 About

AS-Quant is a computational tool used to detect alternative splicing (AS) events between two biological conditions- specifically, two groups of samples- from RNA-seq data. It can categorize five major types of AS in a comparative and comprehensive manner. AS-Quant also includes a visualization tool which generates plots for both the AS events and the annotation of the whole gene.

2 Download

AS-Quant tool can be downloaded directly from https://github.com/CompbioLabUCF/AS-Quant. Users need to have Python installed on their machine. It can work on the Windows, Linux and Mac platforms.

3 Required tools

1. Python (version 3.0 or higher)
2. Samtools 0.1.8* [This specific version]

Required python packages

- matplotlib
  Example Python command: $ sudo apt-get install python3-matplotlib
- scipy
  Example Python command: $ sudo apt-get install python3-scipy
- pandas
  Example Python command: $ sudo apt-get install python3-pandas

4 Run AS-Quant

AS-Quant is designed to work on both human (hg19) and mouse (mm10) alternative splicing events. The supplementary data (the five types of alternative splicing target dataset and the annotation) is provided in the project directory in Github.

Users have to run the following two Python files in order to run AS-Quant:

1. *as_quant.py*: the main function which the users need to run.
2. *make_plots.py*: generates figures for visual representation of data.

4.1 Run as_quant.py

Command: $ **python3 as_quant.py** [**options**]

AS-Quant supports the analysis of multiple samples or replicates (in .bam format) in each group. Users can also select the underlying method [-method option] to determine the significance of each

alternative splicing event. Available methods are: chi-squared test (chisquare) and Wilcoxon rank-sum test (ranksum). If there is more than one sample or replicate in each group, Wilcoxon rank-sum test is an additional option to determine the significance of the events.

List of available options:
(refers to a mandatory field)*

-i*	Input directories. The two directories for the two groups of samples in consecutive order.
-s*	Species name: human/mouse
-o	Output directory
-novel	If the user wants to detect both novel and annotated alternative splicing events. Default is annotated only.
-method	Statistical method to determine the significance of the potential alternative splicing events: chisquare/ranksum. Default is chisquare.

as_quant.py will generate several intermediary files in the output directory. After computing the significance of the association between the two conditions, the final results will be written in the spreadsheet named group1_vs_group2.xlsx, with five separate sheets for five different splicing types. The following image shows some of the generated fields in group1_vs_group2.xlsx for event type 'SE':

Chrom	Gene Name	Exon Start	Exon End	p-value	Ratio difference	Absolute Ratio	Chrom region Long	Event
chr3	TPM3	90091012	90091091	1.59378E-49	-0.337998721	0.337998721	chr3:TPM3:90091012-90091091	SE
chr9	TPM1	67032465	67032541	3.03007E-47	0.172740555	0.172740555	chr9:TPM1:67032465-67032541	SE
chr6	IMMT	71866725	71866740	1.78629E-33	0.339831533	0.339831533	chr6:IMMT:71866725-71866740	SE
chr19	GANAB	8907850	8907916	7.00191E-25	-0.300771862	0.300771862	chr19:GANAB:8907850-8907916	SE
chr2	RBM39	156178879	156178952	9.58374E-24	0.218410281	0.218410281	chr2:RBM39:156178879-156178952	SE
chr10	RPL41	128548657	128548680	9.80399E-19	-0.045526444	0.045526444	chr10:RPL41:128548657-128548680	SE
chr15	PCBP2	102488775	102488814	1.04201E-18	0.095344576	0.095344576	chr15:PCBP2:102488775-102488814	SE
chr6	PHB2	124716424	124716430	7.44053E-18	-0.148651899	0.148651899	chr6:PHB2:124716424-124716430	SE
chr14	COMMD6	101640287	101640299	1.01299E-16	-0.444858768	0.444858768	chr14:COMMD6:101640287-10164029	SE
chr17	TCP1	12917797	12917883	1.35496E-16	0.109515516	0.109515516	chr17:TCP1:12917797-12917883	SE
chr1	FN1	71603659	71603929	1.55266E-16	0.107391529	0.107391529	chr1:FN1:71603659-71603929	SE
chr10	PTBP1	79860116	79860194	1.0884E-15	0.122026419	0.122026419	chr10:PTBP1:79860116-79860194	SE
chr4	RSRP1	134925776	134925821	6.80935E-15	0.178905209	0.178905209	chr4:RSRP1:134925776-134925821	SE
chr17	BAG6	35142497	35142605	1.67888E-14	0.186912982	0.186912982	chr17:BAG6:35142497-35142605	SE
chr9	SNX14	88400722	88400749	9.89647E-14	-0.490833183	0.490833183	chr9:SNX14:88400722-88400749	SE
chr7	SERPINH1	99351823	99351867	5.41593E-13	0.038975242	0.038975242	chr7:SERPINH1:99351823-99351867	SE
chr17	EHMT2	34905609	34905711	1.08835E-12	0.19051368	0.19051368	chr17:EHMT2:34905609-34905711	SE
chr11	RTN4	29706409	29708770	3.74245E-12	0.107629852	0.107629852	chr11:RTN4:29706409-29708770	SE
chr15	PCBP2	102485947	102486040	5.06984E-12	0.087432388	0.087432388	chr15:PCBP2:102485947-102486040	SE
chr2	RBM39	156177632	156177906	5.32911E-12	0.114285345	0.114285345	chr2:RBM39:156177632-156177906	SE

4.2 Running AS-Quant with provided sample input

We provided sample data **'sample_input_mouse'** in our GitHub repository to test AS-Quant, where group1 and group2 are two directories containing the input bam files.
Command: $ **python3 as_quant.py -s mouse -i sample_input_mouse/group1 sample_input_mouse/group2 -o sample_output**

4.3 Run make_plots.py

AS-Quant provides a visualization tool, *make_plots.py*, which generates plots for both the AS events and the annotation of the whole gene. To run the visualization tool, users need to enter the following

command:

$ **python3 make_plots.py -s species -o output_directory -i input1 input2**

Example: $ python3 make_plots.py -s mouse -i sample_input_mouse/group1 sample_input_mouse/group2 -o annotation_plot

Next, *make_plots.py* will ask the users to enter the region of interest for which they want to generate the annotation plot. The format should be specific: **Chom:GeneName:RegionStart-RegionEnd**

parameter descriptions

Chrom	Name of the chromosome
GeneName	Name of the gene
RegionStart	Starting position of the region
Region End	End position of the region

Example: **chr1:Tceb1:16641724-16643478**

make_plots.py will generate the read coverage plot for the given gene along with the whole annotation plot with all exons information of that gene. The figure below shows an example of the read coverage plot generated by AS-Quant.

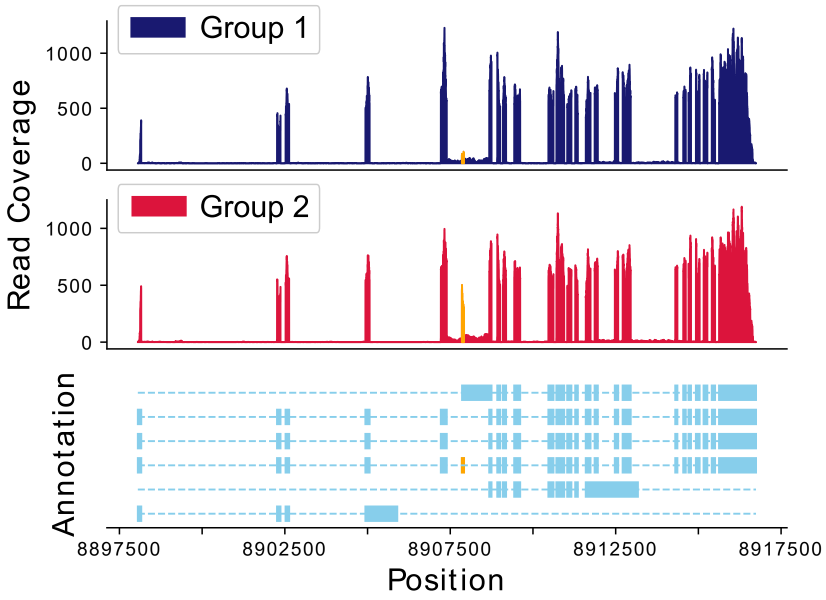

Figure 1: The first two subplots of the figure represent the read coverage of the two biological conditions. The bottom subplot shows the gene annotation and the exon information of that gene.
